# Chondroitin sulfate-E mediates estrogen-induced osteoanabolism

**DOI:** 10.1038/srep08994

**Published:** 2015-03-11

**Authors:** Toshiyasu Koike, Tadahisa Mikami, Miharu Shida, Osami Habuchi, Hiroshi Kitagawa

**Affiliations:** 1 Department of Biochemistry, Kobe Pharmaceutical University, Higashinada-ku, Kobe 658-8558, Japan; 2 Research Complex for the Medical Frontiers, Aichi Medical University, Nagakute, Aichi, 480-1195, Japan

## Abstract

Osteoporosis is an age-related disorder of bone remodeling in which bone resorption outstrips bone matrix deposition. Although anticatabolic agents are frequently used as first-line therapies for osteoporosis, alternative anabolic strategies that can enhance anabolic, osteogenic potential are actively sought. Sex steroid hormones, particularly estrogens, are bidirectional regulators for bone homeostasis; therefore, estrogen-mediated events are important potential targets for such anabolic therapies. Here, we show that estrogen-induced, osteoanabolic effects were mediated via enhanced production of chondroitin sulfate-E (CS-E), which could act as an osteogenic stimulant in our cell-based system. Conversely, estrogen deficiency caused reduced expression of CS-E-synthesizing enzymes, including GalNAc4S-6ST, and led to decreased CS-E production in cultures of bone marrow cells derived from ovariectomized mice. Moreover, *Galnac4s6st*-deficient mice had abnormally low bone mass that resulted from impaired osteoblast differentiation. These results indicated that strategies aimed at boosting CS-E biosynthesis are promising alternative therapies for osteoporosis.

Bone remodeling is a physiological process that maintains skeletal integrity by removing old bone and replacing it with new bone mineral matrix. An imbalance between bone resorption and bone formation causes a number of bone diseases. Osteoporosis, an important and representative age-related disorder of bone remodeling in the modern world, is characterized by enhancement of osteoclastic bone resorption relative to osteoblastic bone formation; this change in the balance leads to bone fragility and increased risk of fractures^1^,^2^,^3^. Currently, anticatabolic agents (e.g., bisphosphonates) are commonly used to prevent osteoporotic bone loss^1^,^4^,^5^. However, such treatments do not always prevent or reduce bone loss in patients, largely because such treatments are not supportive of bone anabolism. Thus, complementary and alternative treatments that cause osteoblastic cells to stimulate bone formation are actively sought^1^,^3^,^6^.

Bone growth and maintenance in both sexes are influenced by sex steroids. Estrogens are major hormonal regulators of bone metabolism, as illustrated by the considerable loss of bone mass in women after natural menopause or oophorectomy^7^. Therefore, estrogen replacement therapy is considered the most effective means for preventing bone loss in women, largely because estrogens inhibit bone resorption^8^. Accumulating evidence also indicates that estrogens are important for skeletal health in men; the bone-protective effects of male estrogens are exerted, at least in part, by promoting osteoblastic differentiation^9^,^10^. Therefore, unraveling the distinctive mechanisms of action during estrogen-mediated bone formation is important for development of more effective anabolic therapies for osteoporosis.

Chondroitin sulfate (CS), a class of sulfated glycosaminoglycans (GAGs), is widely distributed in extra/pericellular matrices in the form of CS proteoglycans (CSPGs); at least one CS side chain of a CSPG is covalently attached to one of a panel of core proteins. CSPGs contribute to physiological milieus that support myriad important cellular events^11^,^12^,^13^,^14^,^15^,^16^,^17^,^18^,^19^,^20^. The wide-ranging biological actions of CSPGs are mainly attributable to the structural complexity of CS moieties^11^,^12^,^15^,^16^,^17^. Each CS moiety comprises repeating disaccharide units of glucuronic acid (GlcA) and *N*-acetylgalactosamine (GalNAc), and can acquire remarkable structural individuality via distinct sulfation modifications^11^,^12^. The abundance and importance of CSPGs in cartilaginous tissues is underscored by the observation that deficiencies of any one of several enzymes that constitute the CS biosynthetic machinery can cause skeletal abnormalities associated with disrupted endochondral ossification^21^,^22^,^23^. Additionally, our previous study demonstrated that CS-E, a CS subtype with characteristic disaccharide E [GlcA-GalNAc(4,6-*O*-disulfate)] units, has multiple distinct roles in osteoblast differentiation^24^. Notably, plasma and tissue levels of CS, like serum levels of estrogens, gradually decrease with age^25^,^26^,^27^,^28^,^29^,^30^. These apparently functional similarities between CS and estrogens indicate that particular CS biosynthetic pathways are critical targets for estrogen-mediated cellular events, including osteoblastic bone formation. Here, we aimed to test whether osteoanabolic activities of estrogens are exerted via CS-E production.

## Results

### Estrogen induces CS-E production in osteoblastic cells

To test our hypothesis, we first used mouse MC3T3-E1 cells to represent osteoblastic cells^31^. MC3T3-E1 cells express N-cadherin and cadherin-11, and cadherin-mediated cell-cell contact is critical for the onset of osteoblastic differentiation of these cells^32^,^33^. Once committed to differentiate, MC3T3-E1 cells produce increasing amounts of CS with a relatively high proportion of E units^24^. Therefore, we treated low-density MC3T3-E1 cultures with estradiol, a predominant form of estrogen, to avoid undesirable CS production that is induced by cell-cell contact. After a 24-h exposure to estradiol, when individual cells remained dispersed, CS isolated from MC3T3-E1 cultures was depolymerized with a bacterial CS-degrading enzyme, chondroitinase ABC (ChABC), and resultant CS disaccharides were analyzed via high-performance liquid chromatography (Table 1). Treatment with estradiol resulted in approximately a 20% increase in the total content of CS disaccharides in low-density cultures; this increase was mainly attributable to elevated levels of 4-*O*-sulfated CS disaccharides, A [GlcA-GalNAc(4-*O*-sulfate)] and E units. In contrast, the amount of 6-*O*-sulfated disaccharide C [GlcA-GalNAc(6-*O*-sulfate)] units was essentially unaltered. This trend was quite similar to that obtained in MC3T3-E1 cultures undergoing differentiation induced by cell-cell contact^24^.

**Figure 1:**
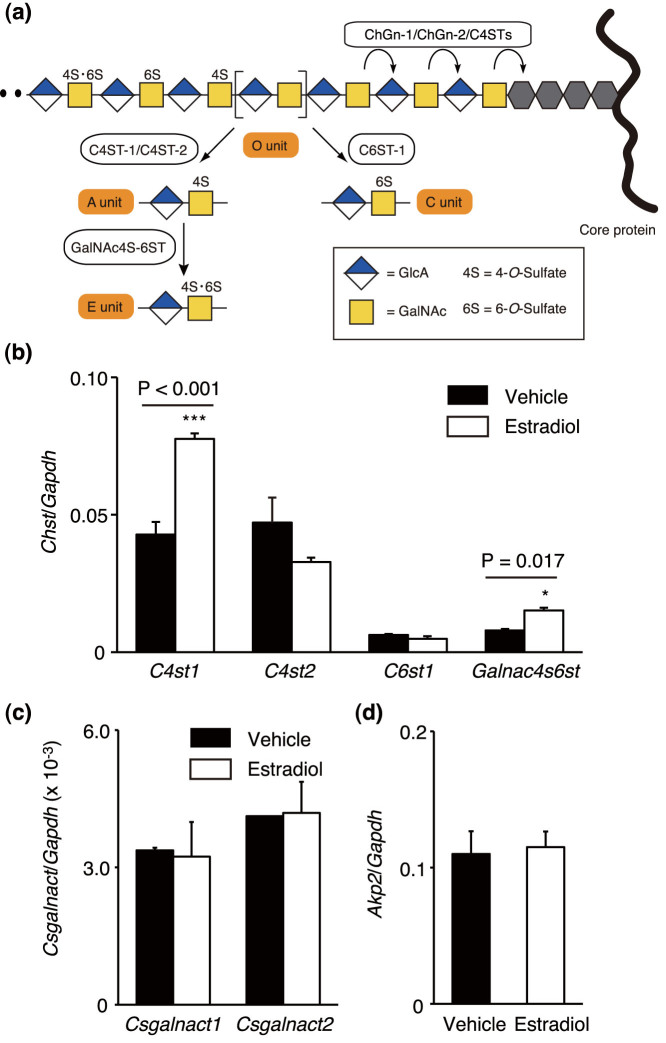
Estradiol promotes CS-E production in osteoblastic cells. (a) Schematic diagram of pathways for sulfation of CS. Characteristic CS disaccharide units including A, C, and E are sequentially formed under the control of CS-specific CHSTs such as C4ST-1, C4ST-2, C6ST-1, and GalNAc4S-6ST. Cooperation of C4STs and ChGns fine tunes CS production. (b–d) Expression of mRNAs encoding CHSTs (*C4st1*, *C4st2*, *C6st1*, or *Galnac4s6st* in b), ChGns (*Csgalnact1* or *Csgalnact2* in c), and ALP (*Akp2* in d) in 24-h cultures of MC3T3-E1 cells in the presence (Estradiol) or absence (Vehicle) of estradiol (n = 3 independent experiments). Data are presented as mean ± s.d. *, P < 0.05; ***, P < 0.001.

These sulfated CS disaccharides (A, E, and C units) are formed during CS biosynthesis^12^. Briefly, precursor disaccharide O units [GlcA-GalNAc] in the chondroitin backbone can serve as common acceptor substrates for two types of sulfotransferases (CHSTs), chondroitin 4-*O*-sulfotransferases (C4STs) or chondroitin 6-*O*-sulfotransferase-1 (C6ST-1). C4STs catalyze 4-*O*-sulfation and C6ST-1 catalyzes 6-*O*-sulfation of GalNAc residues to form A or C units, respectively; subsequent sulfation of A units by an additional enzyme, GalNAc4-sulfate 6-*O*-sulfotransferase (GalNAc4S-6ST), generates E units. Therefore, sulfations of a chondroitin backbone can be classified into “4-*O*-sulfation” or “6-*O*-sulfation” pathways (Fig. 1a). To examine whether the apparently exclusive increase in 4-*O*-sulfated CS containing E units in the estrogen-treated cultures resulted from alteration of CS biosynthetic machinery, expression levels of genes encoding the abovementioned CHSTs were measured. Consistent with the biochemical data for CS, expression of two genes, *C4st1* and *Galnac4s6st*, that encode enzymes involved in 4-*O*-sulfation pathway was significantly upregulated, whereas gene expression relevant to 6-*O*-sulfation was not affected by estradiol treatment (Fig. 1b). Additionally, expression of transcripts encoding ChGn-1 and ChGn-2 did not change significantly (Fig. 1a,c); these enzymes, along with C4STs, catalyze synthesis of the chondroitin backbone and control CS levels^34^,^35^. These findings indicated that the estradiol-induced increases in CS were ascribable to augmentation of *C4st1* expression. Notably, basal expression of *Akp2*, a typical osteogenic marker gene encoding alkaline phosphatase (ALP), remained constant in the 24-h cultures, even in the presence of estradiol (Fig. 1d). Thus, the dynamic changes in expression of genes encoding CS biosynthetic enzymes were not due to secondary effects associated with accelerated differentiation of MC3T3-E1 cells, but instead to possible direct regulation by estradiol.

To examine the generality of the observed phenomena, we examined bone marrow-derived stromal cells (BMSCs) instead of MC3T3-E1 cells, because BMSCs have a significant population of osteoblast progenitors that can contribute to bone formation *in vivo*. In low-density cultures of BMSCs, bath-applied estradiol resulted in a similar pattern of changes in expression of mRNA encoding CHSTs (Supplementary Fig. S1). Thus, these results suggested that estrogen preferentially induces the expression of distinct CS subtypes that have a relatively high amount of E units (hereafter CS-Es) in osteoblastic cells.

### Reduced estrogen level causes reduced CS-E production

**Figure 2:**
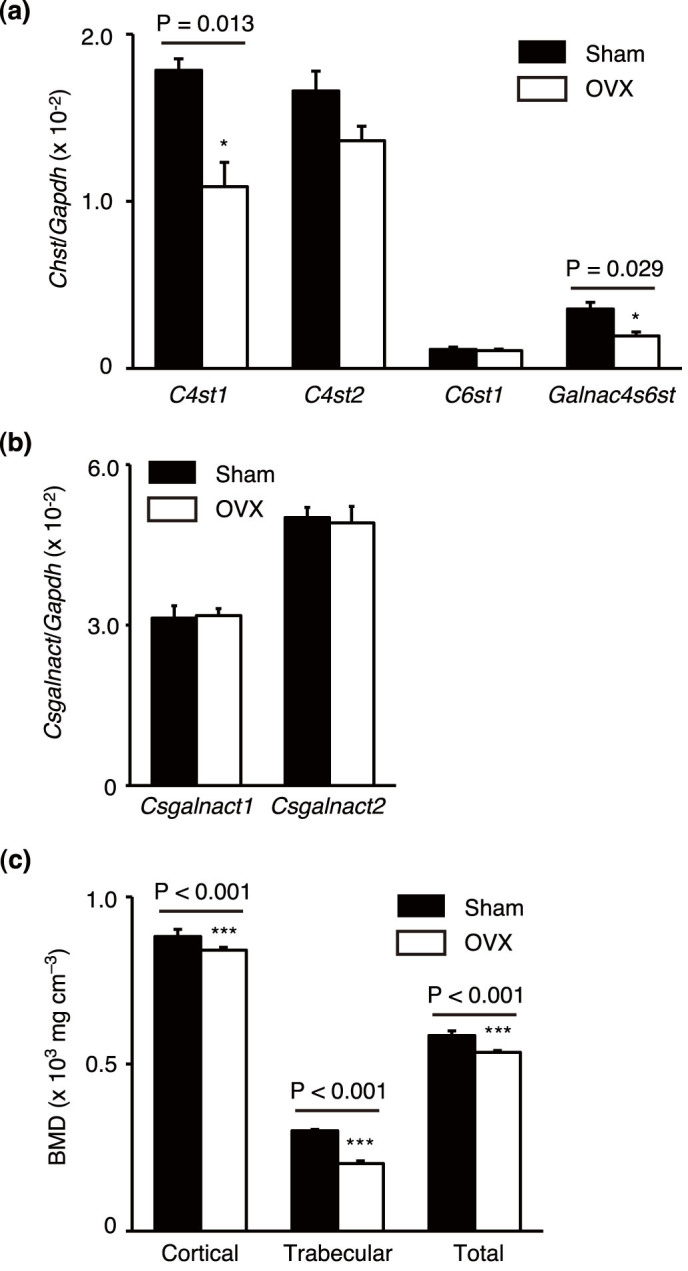
Biosynthetic machinery for CS-E production is affected by estrogen depletion. (a,b) Expression of mRNAs encoding CHSTs (*C4st1*, *C4st2*, *C6st1*, or *Galnac4s6st* in a), and ChGns (*Csgalnact1* or *Csgalnact2* in b) in BMSCs isolated from 14-week-old WT female mice that were sham-operated (Sham) or ovariectomized (OVX) at 8 weeks of age. (n = 3 cultures, each from an independent mouse). Data are represented as mean ± s.d. *, P < 0.05. (c) μCT analysis of tibias of mice in a (n = 3 mice per group). BMD, bone mineral density. Data are represented as mean ± s.d. ***, P < 0.001.

To evaluate *in vivo* effects of estrogens on CS-E production in bone-anabolic milieus, female mice were ovariextomized (OVX) to deplete endogenous estrogens. At 6 weeks after ovariectomy, BMSCs were isolated from femora of either OVX or sham-operated mice, and CS production in the isolated BMSCs was measured. Compared to cells isolated from sham-operated controls (Sham), BMSCs derived from OVX mice exhibited approximately a 50% reduction in CS level and a prominent decrease in the amount of E units (Table 2). Consistent with this observation, levels of *C4st1* and *Galnac4s6st*, genes encoding E-unit-synthesizing enzymes, were significantly lower in BMSCs from OVX mice than those from Sham mice (Fig. 2a). In contrast, ovariectomy did not affect expression of *Csgalnact1* or *Csgalnact2* (Fig. 2b); these findings were reminiscent of those from estradiol-treated MC3T3-E1 cultures (Fig. 1b,c and Supplementary Fig. S1). Additionally, microcomputed tomography (μCT) analysis of tibias, which were excised from respective mice in the same time window as BMSC isolation, was performed to confirm that OVX mice exhibited characteristics of mouse-modeled postmenopausal osteoporosis. Indeed, OVX mice had significantly lower bone mineral density (BMD) in both cortical and trabecular bones than did control mice (Fig. 2c). These findings indicated that CS-E production in bone-forming cells was strictly regulated by estrogens *in vivo*.

### Mice deficient in GalNAc4S-6ST have low bone mass

Based on our notion that CS-E production is a critical event for estrogen-mediated bone anabolism, we expected that genetic deficiencies of C4ST-1 and GalNAc4S-6ST, two enzymes involved in the biosynthesis of E units, would have osteopenic/osteoporotic phenotypes. A gene-trap mutation in *C4st1* causes severe skeletal phenotypes in mice^22^; nevertheless, direct effects of this mutation on bone formation might be difficult to assess because the mutation causes marked chondrodysplasia phenotypes and neonatal lethality. Interestingly, mice lacking GalNAc4S-6ST (*Galnac4s6st^−/−^*) that result in a complete loss of CS-E disaccharides show no readily apparent developmental abnormalities^36^, but detailed phenotypic analyses of *Galnac4s6st^−/−^*mouse skeletons are not published. Therefore, we carefully documented detailed skeletal phenotypes of *Galnac4s6st^−/−^* mice.

**Figure 3:**
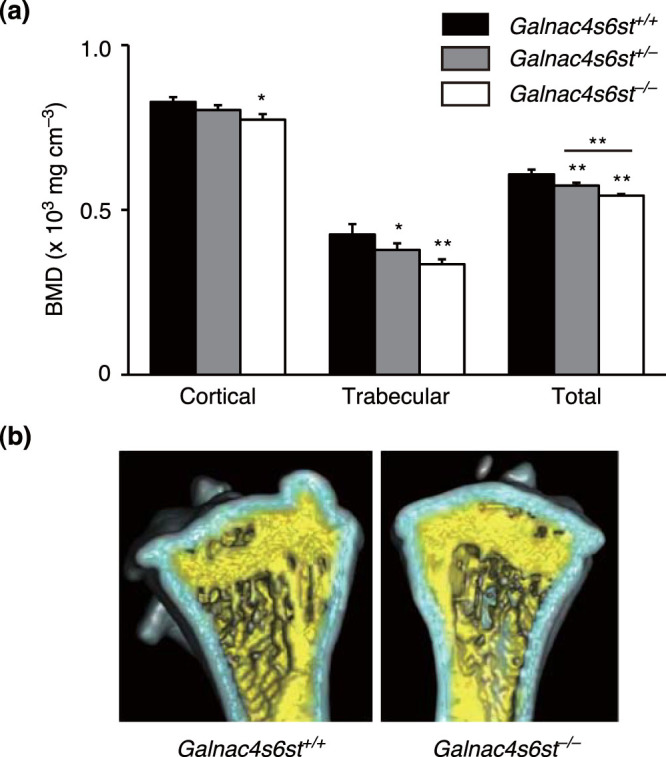
Osteopenic/osteoporotic phenotypes of *Galnac4s6st^−/−^* mice. (a,b) μCT analysis of tibias from 16-week-old male *Galnac4s6st^+/+^* (WT), *Galnac4s6st^+/−^*, or *Galnac4s6st^−/−^* mice. μ-CT-derived measurement of BMD (a, n = 3 bones total, each from different litters). Data are represented as mean ± s.d. *, P < 0.05; **, P < 0.01, Tukey-Kramer test. Medial, longitudinal section through a μCT-generated three-dimensional reconstruction of a tibia (b). The cortical and trabecular regions are pseudo-colored light blue and yellow, respectively.

At 16 weeks after birth, male *Galnac4s6st^−/−^* mice had significantly lower BMD than male wild-type (WT, *Galnac4s6st^+/+^*) littermates; a significant difference between *Galnac4s6st^+/−^* and *Galnac4s6st^+/+^* littermates was also observed in the total BMD (Fig. 3a). Uniform decrease in BMD along the tibia of *Galnac4s6st^−/−^* mice indicated that both cortical and trabecular bones were affected equally (Supplementary Fig. S2). Using μCT three-dimensional reconstruction of tibias, we confirmed these observations (Fig. 3b). Thus, impaired synthesis of CS-E caused both cortical and trabecular bone loss in adult (at least 16-week-old), male mice.

### Regulatory roles of CS-E in bone remodeling

**Figure 4:**
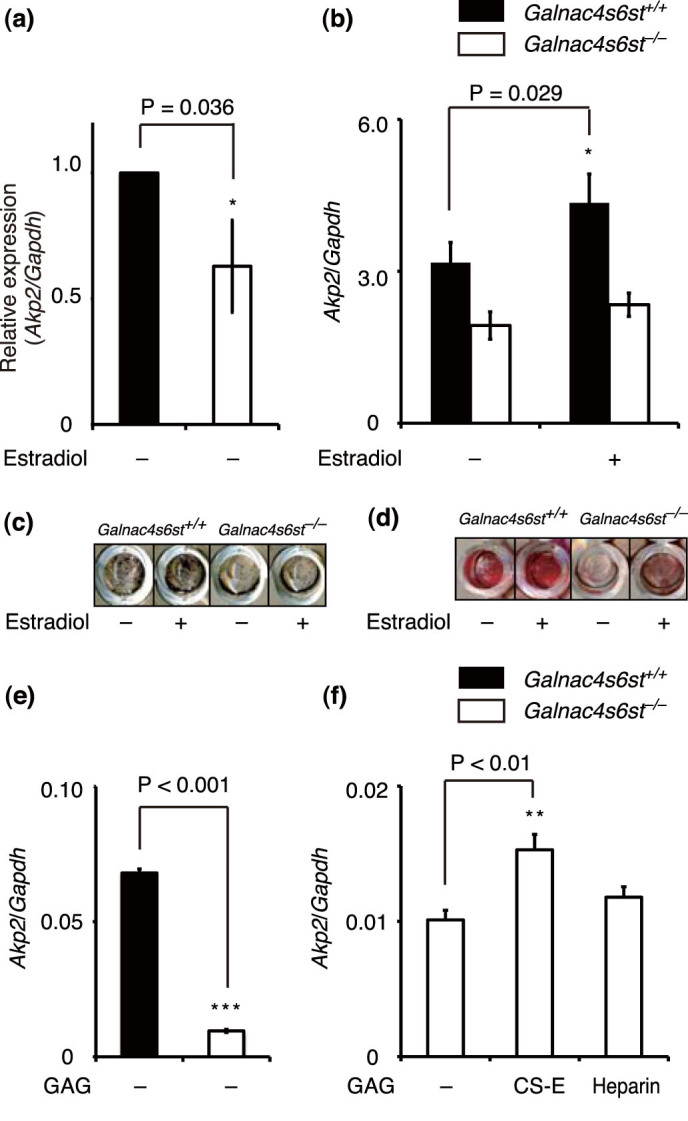
Osteoblastic potential of BMSCs isolated from *Galnac4s6st^+/+^* or *Galnac4s6st^−/−^* mice. (a,b) Expression of *Akp2* mRNA in BMSCs cultured in a differentiation medium (DM) for 4 days in the presence or absence of estradiol (n = 3 cultures total, each from different litters). Data are represented as mean ± s.d. *, P < 0.05. (c,d) BMSCs were maintained in DM for 21 days and stained for ALP after 4 days of culture (c). Alizarin red staining was used to assess mineralized nodule formation (d). (e,f) Expression of *Akp2* mRNA in 24-h cultures of BMSCs in the presence or absence of GAGs, CS-E or heparin (20 μg/ml each). (n = 3 cultures total, each from different litters). Data are presented as mean ± s.d. **, P < 0.01; ***, P < 0.001, Dunnett's test in f.

To examine whether the osteopenic/osteoporotic phenotypes of *Galnac4s6st^−/−^* mice were caused by defects in bone anabolism, the osteoblastic potential of BMSCs, derived either from WT or *Galnac4s6st^−/−^* mice, were assessed. As reported previously^36^, we confirmed that *Galnac4s6st^−/−^* BMSCs also produced CS completely devoid of E units (Table 3). Osteoblast differentiation and maturation constitute a highly ordered process that begins with ALP expression and ends with mineral deposition; therefore, isolated BMSCs were cultured in an osteogenic medium; on days 4 and 21, cells were stained for ALP and mineralized nodule formation, respectively. ALP expression was significantly lower in *Galnac4s6st^−/−^* BMSC cultures than in WT controls (Fig. 4a,c). Mineralized nodule formation in *Galnac4s6st^−/−^* BMSC cultures was also severely impaired (Fig. 4d). Notably, bath application of estradiol led to a significant increase in ALP expression and mineral deposition in WT BMSC cultures, but not in *Galnac4s6st^−/−^* BMSC cultures (Fig. 4a–d). We previously showed that exogenous addition of CS-E polysaccharides could increase ALP expression in the low-density MC3T3-E1 cultures^24^, and such a stimulatory effect was not compensated by heparin, another class of highly sulfated GAGs (Supplementary Fig. S3). Consistent with these findings, bath-applied CS-E, but not heparin, significantly augmented ALP expression even in initial 24-h cultures of *Galnac4s6st^−/−^* BMSCs (Fig. 4f), although the ALP level remained lower than that obtained from intact *Galnac4s6st^+/+^*BMSCs cultures (Fig. 4e,f). These data indicated that CS-E formed by GalNAc4S-6ST is essential for osteoblast differentiation and maturation, and that estrogens are potent inducers of CS-E-mediated osteoblastgenesis.

We also evaluated the osteoclastic potential of bone marrow-derived macrophages (BMMs) isolated from femoral and tibial long bones of WT or *Galnac4s6st^−/−^* mice. In the presence of RANKL (receptor-activator of NF-κB ligand), an osteoclast differentiation factor, BMMs can differentiate into tartrate-resistant acid phosphatase (TRAP)-positive, multinucleated osteoclasts *in vitro*. Based on indexes of osteoclast fusion, we found no significant differences between WT and *Galnac4s6st^−/−^* BMM cultures (Supplementary Fig. S4). This result excluded the possibility that increased osteoclastgenesis in *Galnac4s6st^−/−^* mice might lead to the osteopenic phenotypes, and strengthens the hypotheses that CS-E contributes substantially to bone formation, but not to bone resorption.

## Discussion

Estrogens exhibit pleiotrophic effects on bone homeostasis including bone formation and resorption^9^,^10^. In the present study, we investigated distinctive downstream events of estrogen-mediated osteoanabolism. Our findings demonstrate that CS-E production was critical to proper bone anabolism and that this CS-E production was regulated by estrogens. *Galnac4s6st^−/−^* mice exhibited an undetectable CS-E expression in the bone marrow niche, and experienced significant bone loss because of impaired osteoblastic potential. These findings led to the novel idea that postmenopausal osteopenia and osteoporosis are caused by both excessive osteoclast formation and reduced anabolic bone formation, and that this anabolic activity was mediated by estrogen-induced CS-E. Our previous findings demonstrate that CS-E promotes osteoblast differentiation of MC3T3-E1 cells via its binding to cell surface N-cadherin and cadherin-11^24^. Additionally, osteoblast- and osteocyte-specific conditional knockout of *Cdh2* (N-cadherin) and double *Cdh2^+/−^; Cdh11^−/−^* mutation reportedly lead to osteopenia in adult mice^37^. Hence, estrogen-induced CS-E may also exert anabolic effects on living bone tissue via distinct cadherin-mediated signaling pathways. Since CS production is also up-regulated by cadherin-mediated cell-cell contact^24^, such an initial stimulation of cadherin pathways by estrogen-induced CS-E may induce subsequent CS production to form optimal osteoanabolic milieus. Notably, bath-applied CS-E can activate intracellular signaling required for osteogenesis of MC3T3-E1 cells even in low-density cultures, where cadherin-dependent cell-cell contact does not occur; moreover, these CS-E effects are abrogated by blocking the respective cadherins^24^. Likewise, in *Galnac4s6st^−/−^* BMSCs, exogenous CS-E could also stimulate initial onset of osteoblast differentiation. These findings suggest a mode of action for CS-E; specifically, CS-E may act as functional ligands for two cell surface CS receptors, N-cadherin and cadherin-11. Therefore, CS-E may be useful as pharmacological agents that promote osteogenesis in patients suffering from metabolic bone diseases, including osteopenia and osteoporosis. In addition to CS-E, CS-A level was also up-regulated in estrogen-treated osteoblastic cultures. In contrast, it was dramatically decreased in BMSCs obtained from OVX and *Galnac4s6st^−/−^* mice. In view of the apparent similarities of expression changes in CS-E and CS-A, 4-*O*-sulfated CS-A could also play roles in anabolic bone formation, although, unlike CS-E, exogenous CS-A does not have stimulatory effects on osteogenesis of MC3T3-E1 cultures^24^.

Estrogen replacement therapy is a widely accepted treatment for osteoporosis^8^. However, estrogens have multiple sites of action; consequently, hormonal therapy can increase the risk of thromboembolic events and breast and uterine cancers; accordingly, long-term use of estrogen replacement therapy is limited^38^. To overcome this problem, tissue-targeted hormonal replacement has been proposed. Additionally, strict control of events downstream of estrogens (tissue-specific, function-specific, or both) may become effective therapeutic approaches. In this context, regulation of CS-E expression in bone metabolism may have substantial therapeutic potential. Nevertheless, how CS-E production is controlled by estrogens remains unknown, and direct or indirect transcriptional activation of the E-unit synthesizing enzymes via estrogen receptors is expected to be involved in the process. Furthermore, since CS chains are expressed as CSPGs, identification of core proteins carrying CS-E in the bone marrow niche is also an essential task. Interestingly, double knockout of *Bgn* (biglycan) and *Dcn* (decorin), members of class I type small leucine-rich PGs, results in reduced bone formation and shows osteoporosis-like phenotypes^39^. Therefore, the underlying mechanisms for functional expression of E-unit synthesizing enzymes and the core protein candidates, and the cell and/or tissue specificity of such mechanisms must be clarified for development of CS-E-based therapies. In conclusion, therapies that enhance CS-E production could become highly effective treatments that ameliorate osteopenic/osteoporotic bone loss. To this end, development of small compounds that directly stimulate biosynthetic enzymes that produce CS-E is also highly attractive.

## Methods

### Materials

CS-E polysaccharides from squid cartilage, and *Proteus vulgaris* chondroitinase ABC (EC 4.2.2.4) were purchased from Seikagaku Corp. (Tokyo, Japan). β-Estradiol, and heparin from porcine intestinal mucosa were purchased from Sigma.

### Mice

Mice (C57BL/6) including *Galnac4s6st^−/−^* (Ref. 36) mice were kept under pathogen-free conditions in an environmentally controlled, clean room at the Institute of Laboratory Animals, Kobe Pharmaceutical University; animals were maintained on standard rodent food and on a 12-h light/12-h dark cycle.

Eight-week-old virgin female WT mice were subject to either bilateral sham operation or ovariectomy and then reared for 6 weeks before use.

### Osteoblast cultures

Mouse osteoblastic MC3T3-E1 cells (RCB1126) were obtained from RIKEN Cell Bank (Tsukuba, Japan), and these at passages 3–10 were used in this study. BMSCs were harvested from long bones (femora plus tibias or femora alone) of 14-week-old WT female mice from the intact, sham-operated, or ovariectomized group and from femora of 16-week-old WT or *Galnac4s6st^−/−^* male mice as described^40^. Based on the representative flow cytometric profiles (Supplementary Fig. S5), the isolated BMSCs were largely free of contamination of endothelial and hematopoietic cells. Mouse osteoblastic MC3T3-E1 cells or BMSCs from WT intact female mice were seeded at a low density (5 × 10^3^ cells cm^−2^) and incubated for 24 h in growth medium (GM, αMEM containing 10% FBS) in the presence or absence of 10 nM estradiol. To assess the influence of estrogen deficiency, BMSCs from sham-operated or ovariectomized WT female mice were cultured in GM for 3 days. BMSCs from WT or *Galnac4s6st^−/−^* male mice were placed on 96 well plates at a high density (1 × 10^4^ cells per well) and cultured for 21 days in differentiation medium (DM, GM supplemented with 100 μg ml^−1^ ascorbic acid, 10 mM β-glycerophosphate and 10 nM dexamethasone) to elicit osteoblast differentiation^24^. In some cases, BMSCs were maintained in DM supplemented with 10 nM estradiol for 4 days. ALP staining^24^ for cellular ALP activity and alizarin red staining for measurement of mineral matrix formation were performed at days 4 and 21, respectively. To examine the effects of exogenous GAGs on osteoblast differentiation, low-density cultures of MC3T3-E1 cells or *Galnac4s6st^−/−^* BMSCs were performed for 24 h in GM in the presence or absence of GAGs (CS-E or heparin, 20 μg/ml each).

### Osteoclast cultures

Bone marrow cells were harvested from femora and tibias of 8 to 12-week old WT or *Galnac4s6st^−/−^* male mice. BMMs were concentrated from bone marrow cells as described^41^. To induce osteoclast differentiation, the resultant BMMs (2 × 10^4^ cell per well of 96-well plates) were stimulated with M-CSF (50 ng ml^−1^), TGF-β (1 ng ml^−1^), and RANKL (300 ng ml^−1^) for 72 h. To evaluate osteoclast formation, cells were stained for TRAP^41^.

### Disaccharide composition of CS

CS from MC3T3-E1 and BMSC cultures was prepared as described previously^42^. The purified glycosaminoglycan fraction containing CS was digested with chondroitinase ABC at 37°C for 2 h. The digests were derivatized with the fluorophore 2-aminobenzamide and were then analyzed via anion-exchange HPLC on a PA-03 column (YMC, Kyoto, Japan). Identification and quantification of the resulting disaccharides were achieved by comparison with authentic unsaturated CS disaccharides (Seikagaku).

### Quantitative real-time RT-PCR

TRIzole reagents were used to extract total RNA from osteoblastic cells. An aliquot of each total RNA (1 μg) sample was pretreated with RNase-free DNase to serve as template for cDNA synthesis. FastStart DNA Master plus SYBR Green I and a LightCycler ST300 (Roche) were used to perform quantitative real-time RT-PCR. The following primer sets were used: *Gapdh*, forward primer, 5′–CATCTGAGGGCCCACTG–3′ and reverse primer, 5′–GAGGCCATGTAGGCCATGA–3′; *C4st1*, forward primer, 5′–GCTGGAAGTGATGAGGATGAA–3′ and reverse primer, 5′–GCTGGATGGGATTGTAGAG–3′; *C4st2*, forward primer, 5′–ATCAGCATCACCAGCAACA–3′ and reverse primer, 5′–TTGGTCATGCTGCCCTG–3′; *C6st1*, forward primer, 5′–CTGGCATTTGTGGTCATAGTTT–3′ and reverse primer, 5′–AAGAGAGATGCATTCTCCGATAAG–3′; *Galnac4s6st*, forward primer, 5′–TATGACAACAGCACAGACGG–3′ and reverse primer, 5′–TGCAGATTTATTGGAACTTGCGAA–3′; *Csgalnact1*, forward primer, 5′–TAAACAGCCCTGTGGAGAG–3′ and reverse primer, 5′–GTCGAAATAGGACAAGTCGC–3′; *Csgalnact2*, forward primer, 5′–TTAATATCATTGTGCCACTTGCG–3′ and reverse primer, 5′–TAGAATAGACTTGACTTTAGATAGTCCTT–3′; and *Akp2*, forward primer, 5′–CCTGACTGACCCTTCGC–3′ and reverse primer, 5′–GTCAAGGTGTCTTTCTGGGA–3′. The expression level of each gene was normalized to that of *Gapdh*.

### μCT

BMD assessment and reconstruction of three-dimensional images of tibias from sham-operated or ovariectomized female WT or 16-week-old male *Galnac4s6st^−/−^* mice were conducted using μCT (LaTheta LCT-200, ALOKA).

### Statistical analysis

Unless otherwise specified, statistical significance was determined via two-tailed Student's *t* test. Differences were considered to be significant with a P value less than 0.05.

### Ethics statement

All animal procedures were approved by the Kobe Pharmaceutical University Committee on Animal Research and Ethics. All experiments were conducted in accordance with the institutional ethical guidelines for animal experiments and safety guidelines for gene manipulation experiments.

## Author Contributions

T.K., T.M., M.S. and H.K. designed and performed the research, analyzed the data and wrote the manuscript. H.K. conceived the idea. O.H. produced the *Galnac4s6st^−/−^* mice.

## Supplementary Material

Supplementary Information Supplementary Info

## Figures and Tables

**Table 1:** Disaccharide composition of CS isolated from estradiol-treated or vehicle-treated MC3T3-E1 cells

	pmol/mg (mol%)^b^	
Disaccharides^a^	Vehicle	Estradiol
ΔDi-0S	22.4 ± 3.7 (5.8)	17.3 ± 1.8 (3.8)
ΔDi-6S	6.1 ± 0.9 (1.8)	6.5 ± 1.2 (1.4)
ΔDi-4S	347.0 ± 14.6 (89.8)	415.9 ± 29.7* (91.6)
ΔDi-diS_D_	N.D.^c^	N.D.
ΔDi-diS_E_	10.8 ± 1.7 (2.8)	14.4 ± 1.9** (3.2)
Total	386.3 ± 15.7 (100)	454.0 ± 33.9* (100)

^a^Abbreviations: ΔDi-0S, ΔHexA-GalNAc; ΔDi-6S, ΔHexA-GalNAc(6-*O*-sulfate); ΔDi-4S, ΔHexA-GalNAc(4-*O*-sulfate); ΔDi-diS_D_, ΔHexA(2-*O*-sulfate)-GalNAc(6-*O*-sulfate); ΔDi-diS_E_, ΔHexA-GalNAc(4,6-*O*-disulfate). ΔHexA represent an unsaturated hexuronic acid that was generated via catalysis by bacterial CS-degrading enzymes, including chondroitinase ABC.

^b^The values are expressed as pmol of disaccharide per mg of dried homogenate and represent the mean ± s.d. (n = 4). The values in parentheses represent molar ratio of the disaccharides.

^c^N.D., not detected.

*, P < 0.05;

**, P < 0.01.

**Table 2:** Disaccharide composition of CS from BMSCs derived from sham-operated or ovariectomized female mice

Disaccharides	pmol/mg (mol%)^a^	
	Sham	OVX
ΔDi-0S	4.6 ± 1.5 (0.5)	14.1 ± 1.2** (3.2)
ΔDi-6S	4.9 ± 0.7 (0.6)	2.7 ± 0.8* (0.6)
ΔDi-4S	799.4 ± 9.8 (95.0)	417.7 ± 15.3*** (94.5)
ΔDi-diS_D_	N.D.	N.D.
ΔDi-diS_E_	32.5 ± 2.1 (3.9)	7.5 ± 1.3*** (1.7)
Total	841.4 ± 8.8 (100)	442.0 ± 16.3*** (100)

^a^The values represent the mean ± s.d. (n = 3).

*, P < 0.05;

**, P < 0.01;

***, P < 0.001.

**Table 3:** Disaccharide composition of CS isolated from *Galnac4s6st^+/+^* or *Galnac4s6st^−/−^* mice

	pmol/mg (mol%)^a^	
Disaccharides	*Galnac4s6st^+/+^*	*Galnac4s6st^−/−^*
ΔDi-0S	40.7 ± 8.8 (5.4)	20.2 ± 1.2 (5.2)
ΔDi-6S	7.0 ± 1.1 (0.9)	4.1 ± 0.2* (1.1)
ΔDi-4S	692.7 ± 38.6 (91.7)	361.5 ± 18.4*** (93.7)
ΔDi-diS_D_	N.D.	N.D.
ΔDi-diS_E_	15.0 ± 7.1 (2.0)	N.D
Total	755.4 ± 39.7 (100)	385.8 ± 17.5*** (100)

^a^The values represent the mean ± s.d. (n = 3).

*, P < 0.05;

***, P < 0.001.
