# Supplementary material for: Chondroitin sulfate-E mediates estrogen-induced osteoanabolism
Source: Sci Rep. 2015 Mar 11;5:8994. doi: 10.1038/srep08994 (PMC4355730; doi:10.1038/srep08994)
Supplement: Supplementary Information — Supplementary Info [file srep08994-s1.pdf]

Supplementary information

Chondroitin sulfate-E mediates estrogen-induced osteoanabolism.

Toshiyasu Koike[1], Tadahisa Mikami[1], Miharu Shida[1], Osami Habuchi[2] & Hiroshi Kitagawa[1*]

[1]Department of Biochemistry, Kobe Pharmaceutical University, Higashinada-ku, Kobe 658-8558, Japan. [2]Research Complex for the Medical Frontiers, Aichi Medical University, Nagakute, Aichi, 480-1195, Japan.

*Correspondence and requests for materials should be addressed to H.K. (kitagawa@kobepharma-u.ac.jp).

Supplementary methods

Flow cytometry. The cultured BMSCs were detached by Accutase treatment, and washed with PBS containing 3% FBS and 0.1% NaN_3 (FACS buffer). The single cell suspensions (1.5 x 10^4 cells/test) were incubated with fluorescein isothiocyanate (FITC)-conjugated anti-mouse Sca-1 (stem cell antigen-1, eBioscience, 1:100), phycoerythrin (PE)-conjugated anti-mouse CD31 (eBioscience, 1:100), or PE-conjugated anti-mouse CD45 (eBioscience, 1:100) antibody for 1 h on ice. After washing with FACS buffer, cells were analyzed by BD Accuri™ C6 flow cytometer (BD Bioscience).

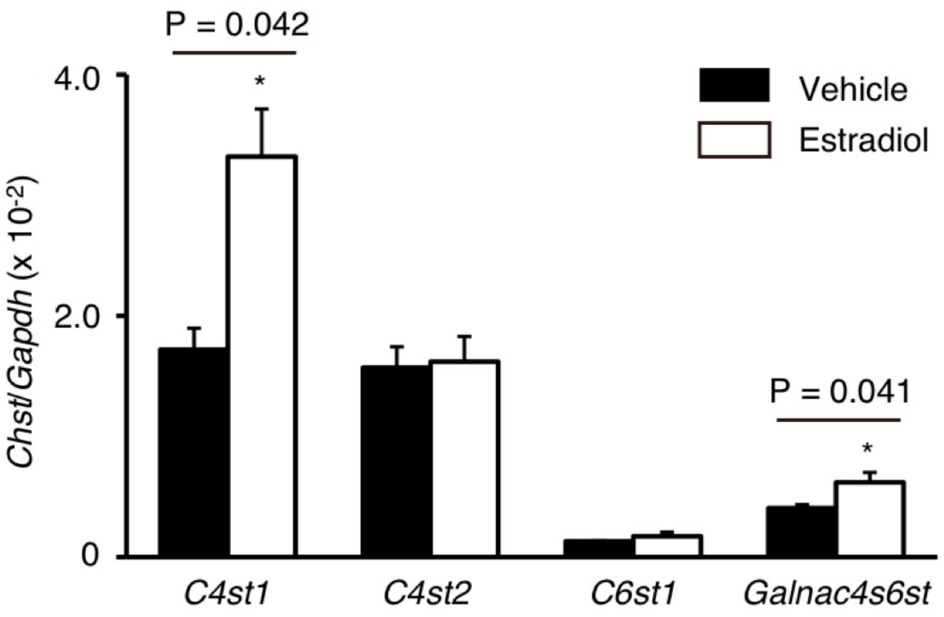

Supplementary Figure S1 | Estradiol augments expression of CS-E-synthesizing enzymes in BMSCs. Expression of mRNAs encoding CHSTs (*C4st1*, *C4st2*, *C6st1*, or *Galnac4s6st*) in BMSCs isolated from 14-week-old WT female mice. (n = 3 cultures, each from an independent mouse). Data are represented as mean ± s.d. *, $P < 0.05$.

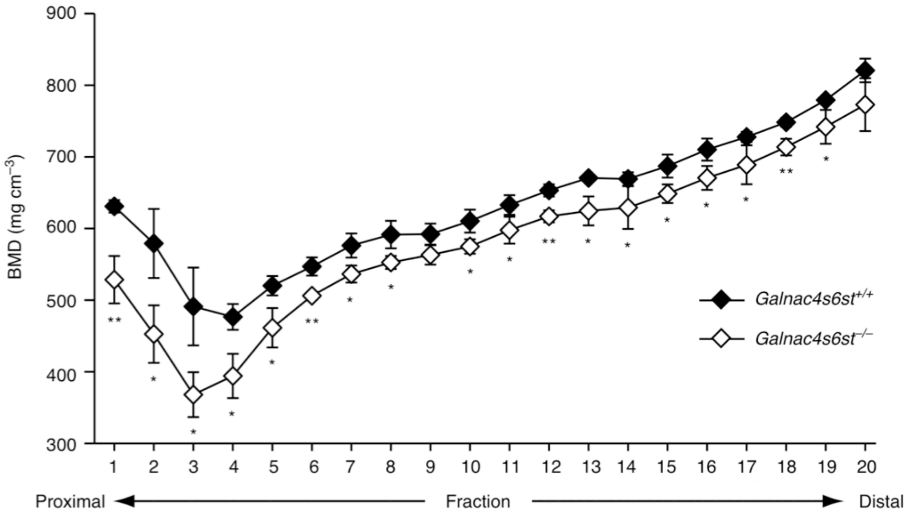

Supplementary Figure S2 | Bone mass in tibias of *Galnac4s6st*$^{-/-}$ mice. BMD of each of 20 equal longitudinal divisions of tibias from *Galnac4s6st*$^{+/+}$ or *Galnac4s6st*$^{-/-}$ mice (n = 3 bones total, each from different litters). Data are represented as mean ± s.d. *, $P < 0.05$; **, $P < 0.01$.

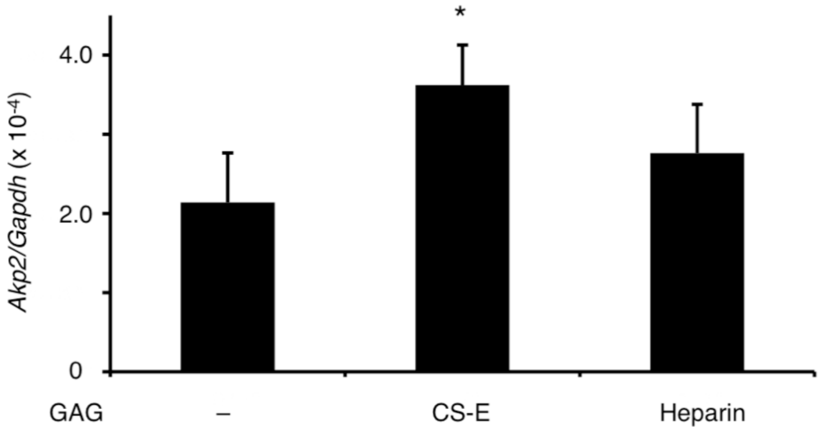

Supplementary Figure S3 | Exogenous CS-E, but not heparin, induces ALP expression in the low-density MC3T3-E1 cultures. Expression of *Akp2* mRNA in the low density MC3T3-E1 cultures in GM for 24 h in the presence or absence of GAGs (CS-E or heparin, 20 µg/ml each) (n = 3 independent experiments). Data are represented as mean ± s.d. *, $P < 0.05$, Dunnett's test.

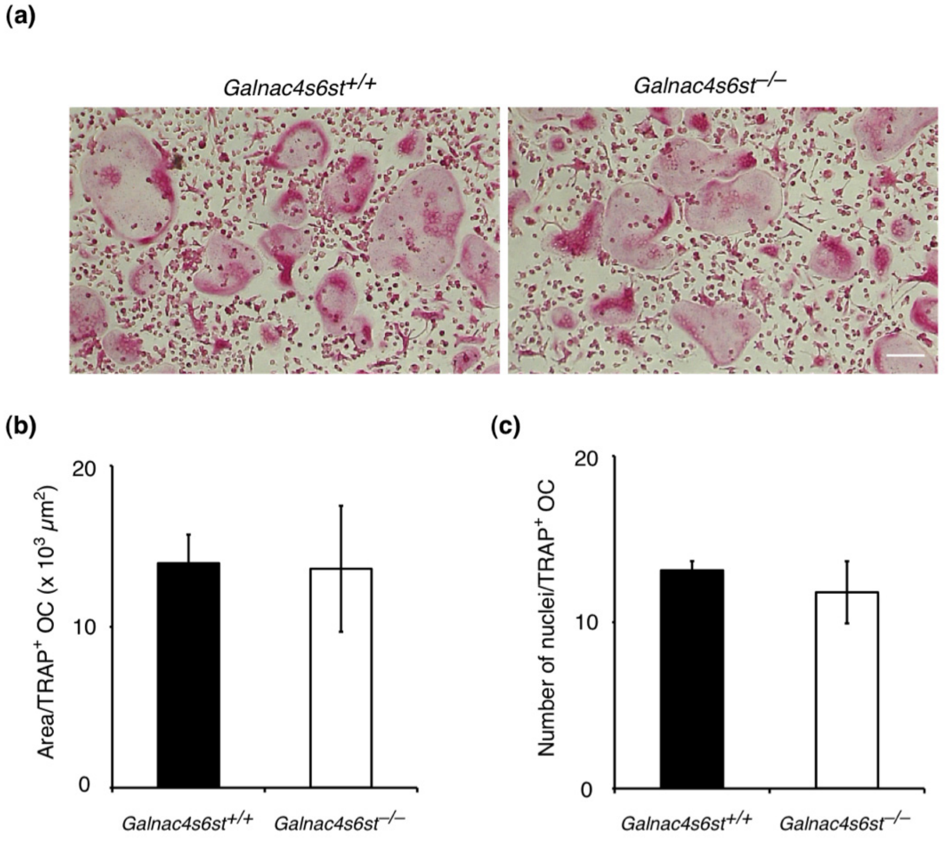

Supplementary Figure S4 | Osteoclast fusion is apparently normal in *in vitro* cultures of BMMs derived from *Galnac4s6st*$^{-/-}$ mice. BMMs derived from *Galnac4s6st*$^{+/+}$ or *Galnac4s6st*$^{-/-}$ mice were cultured for 72-h in DM supplemented with RANKL to facilitate osteoclastgenesis. TRAP-stained osteoclastic cells (OCs) (a), mean area per OC (b), and the number of nuclei per OC (c) are shown as indications of osteoclastgenesis (n = 3 cultures, each from different litters). Data are represented as mean ± s.d. Scale bar in a, 100 μm.

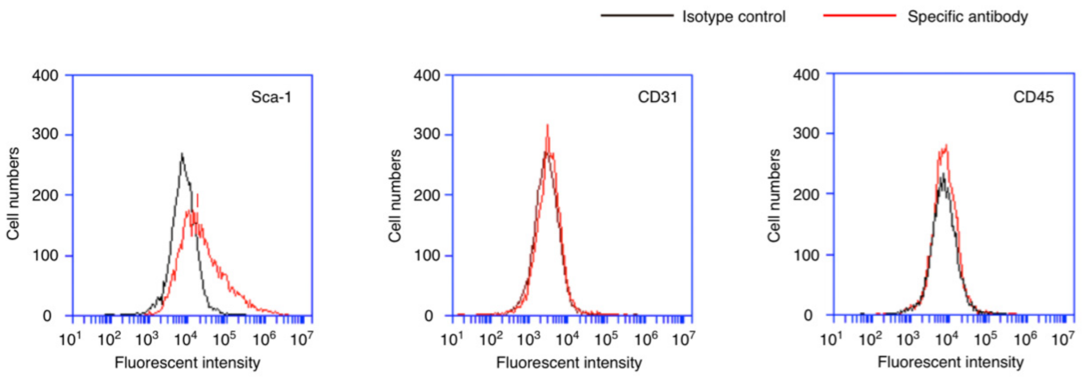

Supplementary Figure S5 | Immunophenotypic characteristics of BMSCs used in this study. The isolated WT BMSCs were labeled with FITC-conjugated anti-Sca-1 (a mesenchymal stem cell marker), PE-conjugated anti-CD31 (an endothelial cell marker), or PE-conjugated anti-CD45 (a hematopoietic marker) antibody. Flow cytometric analysis showed that the cultured cells were positive for a typical mesenchymal stem cell marker, but negative for endothelial and hematopoietic markers.
